# Supplementary material for: The Construction and Exploration of a Comprehensive MicroRNA Centered Regulatory Network in Foxtail Millet (Setaria italica L.)
Source: Front Plant Sci. 2022 May 6;13:848474. doi: 10.3389/fpls.2022.848474 (PMC9121102; doi:10.3389/fpls.2022.848474)
Supplement: Supplementary file 4 [file Data_Sheet_4.zip › Sit-miR171g_Seita.2G419600.1_5912_TPlot.pdf]

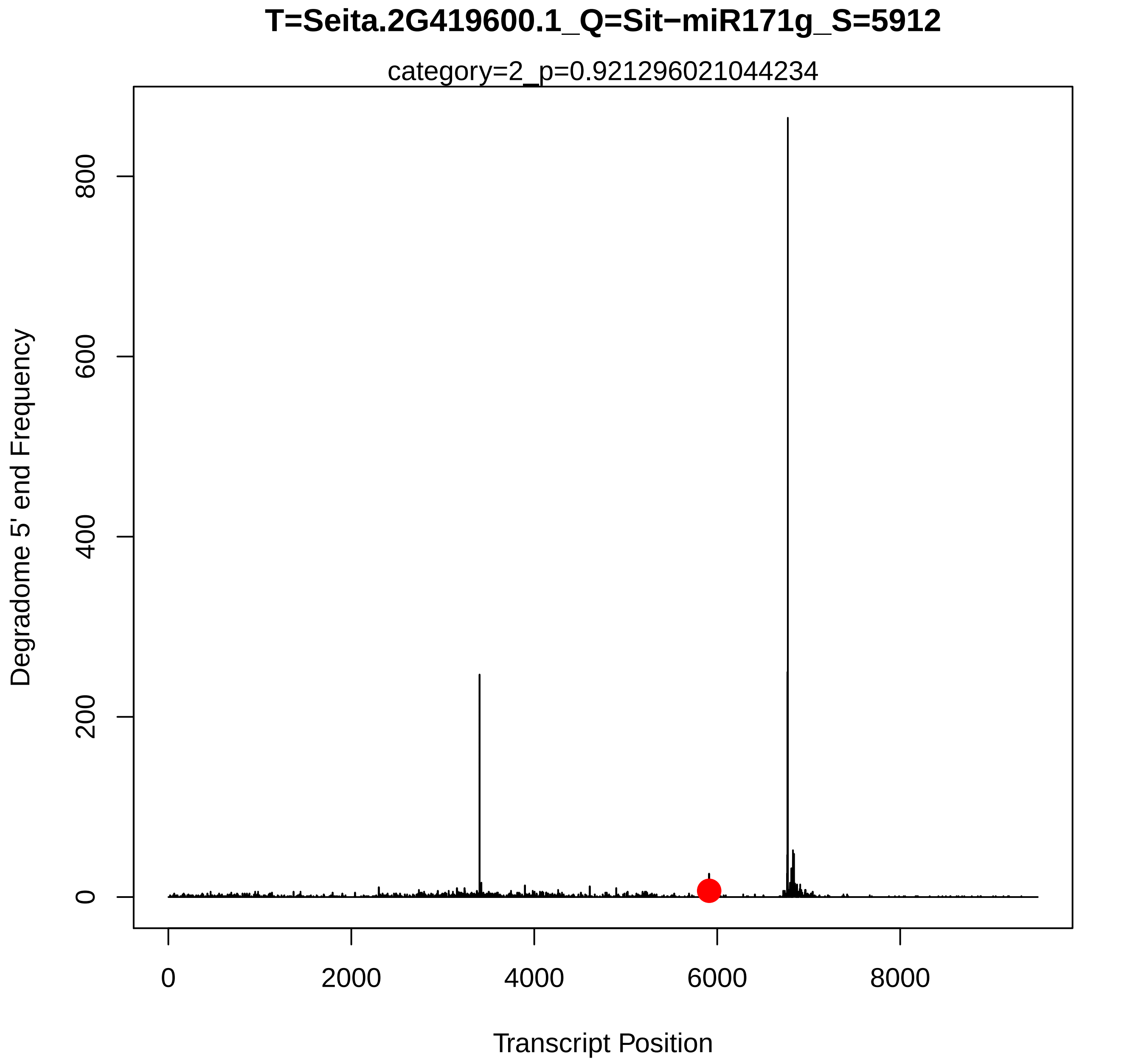

T=Seita.2G419600.1_Q=Sit−miR171g_S=5912
category=2_p=0.921296021044234
Degradome 5' end Frequency
0
200
400
600
800
0
2000
4000
6000
8000
Transcript Position
